# Smart waste bin monitoring using IoT for sustainable biomedical waste management

**DOI:** 10.1007/s11356-023-30240-1

**Published:** 2023-10-25

**Authors:** Aliyu Ishaq, Shamsuddeen Jumande Mohammad, Al-Amin Danladi Bello, Surajo Abubakar Wada, Adejimi Adebayo, Zainab Toyin Jagun

**Affiliations:** 1 Department of Water & Environmental Engineering, School of Civil Engineering, Faculty of Engineering, Universiti Teknologi Malaysia, 81300 Bahru, Johor Malaysia; 2 Department of Water Resources and Environmental Engineering, Ahmadu Bello University, Zaria, Nigeria; 3 Department of Civil Engineering, Ahmadu Bello University, Zaria, Nigeria; 4 Department of Real Estate, School of Built Environment Engineering and Computing, Leeds Beckett University, City Campus Leeds, Leeds, UK

**Keywords:** Biomedical waste, Internet of Things, Monitoring, Smart waste bin, Sustainable, Waste management

## Abstract

Suboptimal management of healthcare waste poses a significant concern that can be effectively tackled by implementing Internet of Things (IoT) solutions to enhance trash monitoring and disposal processes. The potential utilisation of the Internet of Things (IoT) in addressing the requirements associated with biomedical waste management within the Kaduna area was examined. The study included a selection of ten hospitals, chosen based on the criterion of having access to wireless Internet connectivity. The issue of biomedical waste is significant within the healthcare sector since it accounts for a considerable amount of overall waste generation, with estimates ranging from 43.62 to 52.47% across various facilities. Utilisation of (IoT) sensors resulted in the activation of alarms and messages to facilitate the prompt collection of waste. Data collected from these sensors was subjected to analysis to discover patterns and enhance the overall efficiency of waste management practices. The study revealed a positive correlation between the quantity of hospital beds and the daily garbage generated. Notably, hospitals with a higher number of beds were observed to generate a much greater amount of waste per bed. Hazardous waste generated varies by hospital, with one hospital leading in sharps waste (10.98 kgd^−1^) and chemical waste (21.06 kgd^−1^). Other hospitals generate considerable amounts of radioactive waste (0.60 kgd^−1^ and 0.50 kgd^−1^), pharmaceuticals, and genotoxic waste (16.19 kgd^−1^), indicating the need for specialised waste management approaches. The study sheds light on the significance of IoT in efficient waste collection and the need for tailored management of hazardous waste.

## Introduction

Waste management in hospitals has become a grievous threat to the community’s health (Ezeudu et al. 2022) and the health of hospital employees due to its inherent toxic and infectious nature (Behnam et al. 2020). Hospital waste is produced during human or animal diagnosis, treatment, and vaccination. It consists of blood-stained bandages, laboratory implements, disposable gloves and surgical instruments, syringes, tape, etc. (Khalid et al. 2021). Hospital waste can be broadly categorised into hazardous and non-hazardous (Kumarasamy & Jeevaratnam 2017; Attrah et al. 2022). The hazardous waste stream encompasses infectious wastes, such as those contaminated with pathogens (Attrah et al. 2022); (Ezeudu et al. 2022). Chemical wastes result from various medical procedures (Chioma et al. 2019; Shivalli & Sanklapur 2014; Farzadkia et al. 2018), and radioactive wastes originate from diagnostic and therapeutic applications (Palanisamyypasupathi and Athimoolammambika 2021; Rakesh et al. 2023). Laboratory equipment, leftover meals, and discarded fruit are all examples of non-hazardous waste (Rakesh et al. 2023; Behnam et al. 2020). Factors such as hospital type, waste-generating area, waste management practices, hospital specialisation, percentage of reusable items used in hospitals, and patient flow all influence the total and composition of hospital waste (Ogbonna 2013).

In a study of hospital waste generation and management practice in Nigeria, it was observed that there is no uniform practice of hospital waste management among the hospitals (Wahab & Adesanya 2011) studied. All wastes are often mixed in the same wastebasket, posing a cross-contamination risk (Babatola 2018; Wahab & Adesanya 2011). Only about 35% of the hospitals studied segregate their wastes, and most of the hospitals use sanitary landfills as a means of final disposal. Their practice does not follow the recommended standards (Ogbonna et al 2012). Nigerian hospitals lack reliable data on hospital waste’s characterisation and generation rate (Ezeudu et al. 2022; Ogbonna 2013). Most hospitals in Nigeria do not have a proper system for managing hospital waste. The most common methods of hospital waste disposal in Nigeria are landfilling and open burning (Jagun et al. 2022). These methods are not environmentally friendly and can pose a health risk to the public (Ogbonna 2013; Ishaq et al. 2023).

Without proper waste management, hospitals’ large amounts of waste might harm patients’ health (Abdullahi et al. 2015). IoT (Internet of Things) technology can be used for hospital waste management by giving real-time data on waste quantities, types, and locations (Uganya et al. 2022). Waste collection routes can be improved, waste output reduced, and public security bolstered with this data (Dubey et al. 2020). The Internet of Things (IoT) is an emerging technology with the potential to alter the waste collection industry completely. Dubey et al. (2020) argued that Internet of Things–based biomedical solid waste management systems can aid in making hospital waste management more efficient and sustainable.

The Internet of Things concept can be comprehended as an intelligent method of communicating with non-digital entities, encompassing perception, networking, and information processing. This facilitates collaboration among humans, objects, and services within three-layered networks, eliminating the necessity for human intervention (Park et al. 2022; Shirke et al. 2008). The Internet of Things is dependable due to its incorporation with cloud data systems. Automation is advantageous for waste management monitoring systems as it decreases manual labour and enhances operational efficiency (Shirke et al. 2008). The Internet of Things empowers stakeholders to monitor real-time data and obtain insights into the current state of waste (Rhee 2020). The Internet of Things can greatly enhance biomedical waste handling (Singh and Misra 2022). Smart bins with sensors keep track of their capacity and transmit alarms to waste management employees when they reach full capacity (Deepa 2021; Dubey et al. 2020), preventing overflow and associated health problems. Also, only full bins are serviced, reducing fuel consumption and emissions (Mohamed et al. 2023), thanks to the real-time location monitoring made possible by the GPS in the smart bins (Wawale et al. 2022). Moreover, sensors within these bins can distinguish distinct waste categories (Dey et al. 2021; Wang et al. 2020), allowing for efficient segregation at the source, facilitating recycling, composting, and reducing landfill waste (Karnavel et al. 2023; Wang et al. 2020). Moreover, sensor data can be utilised to identify and prosecute perpetrators of fraudulent acts like illegal dumping, aided by IoT-based solutions (Gayathri et al. 2022; Raundale et al. 2018). These Internet of Things–enabled techniques can considerably improve hospital waste management, which benefits sustainability and environmental health (Annan et al. 2022; Karnavel et al. 2023).

The Republic of Korea has recently unveiled a remarkable innovation in waste management—RFID bags. These bags are equipped with radio frequency identification technology, which enables the tracking of waste products from their origin in hospitals to their final destination at disposal sites (Chauhan et al. 2021). The tags on these bags can be scanned, allowing waste contractors to exchange information about the waste effortlessly. This innovative approach extends beyond managing municipal garbage to encompass the effective handling of hospital waste. In contrast, most other nations continue to depend on manual methods for treating hospital waste (Chauhan et al. 2021).

Effective medical waste management involves a range of activities beyond waste disposal, including segregation, collection, transportation, storage, handling, and documentation to ensure the safe handling and disposal of hazardous and infectious materials, safeguarding both humans and the environment (Omoleke et al. 2021). Despite significant technological developments in waste management, numerous systems depend on human labour, which presents various issues. For example, in medical waste management, collection workers have to empty waste bins every 4 h, with the status of waste collection recorded on bin documents shared with management at the end of each day (Oyekale & Oyekale 2017). However, this approach is susceptible to disorganisation and inefficiency and has even led to injuries among waste collectors. The difficulty in accurately segregating waste is further highlighted by the need for advanced and automated waste management systems (Park et al. 2022). Typically, waste is only weighed at storage facilities before generating a consignment note for the transportation company. Monitoring storage conditions is often manual or neglected altogether. Waste is transported along designated routes, but the location of lorries is not consistently tracked, although RFID tags are used in some countries to facilitate access to disposal stations (Rhee 2020).

For waste monitoring, sensors are affixed to the bin lid (Park et al. 2022; Shirke et al. 2008). The sensors are equipped with WiFi or Bluetooth connectivity, enabling the seamless transmission and processing of data acquired by the sensors or actuators in waste monitoring. The data acquired is then stored in the cloud, enabling easy access to the requisite services. In current waste management, geospatial technology tracks transportation routes, vehicles, and waste bins. In some areas, waste disposal may not be very active. To conserve power and enable the connectivity of the smart bin to the Internet, an ultrasonic sensor can be utilised to detect the presence of waste. Employing ultrasonic sensors for waste management is highly beneficial as the weight of waste impacts its size and knowing the weight can be advantageous for storage, transportation, and disposal facilities. In some studies, ultrasonic sensors are utilised to gather data on waste levels in the bin. This information can assist management in planning waste collection and transportation. In most cases, the weight of waste impacts its size. Therefore, knowing the weight can benefit storage, transportation, and disposal facilities. Storage facilities can allocate space accordingly, transportation can identify and prepare suitable vehicles for collection, and disposal facilities can use this information to approximate disposal efficiency. Hussain et al. (2020) employed the HX711 weight sensor to establish a blockchain in medical waste management, demonstrating a creative approach to waste monitoring.

Researchers have proposed several approaches to provide optimal service in waste monitoring using the Internet of Things. Data gathered by sensors are usually linked to microcontrollers like Arduino Uno. To enable optimal service in waste monitoring, researchers have proposed several approaches, including the integration of simple technologies like RFID and QR codes with microcontrollers, RFID readers, and Raspberry Pis. Wawale et al. (2022) have suggested using RFID connected to the IoT via the fuzzy method to monitor waste management. Four questions are posed to the waste before any decision is made. The fuzzy method will ask the scanned items these four questions before categorising them into any bins.

To establish a robust contractual framework among stakeholders, focusing on weighing the waste, Wang et al. (2020) proposed utilising blockchain technology and the QR method. This method can be highly beneficial as it enables stakeholders to track the waste throughout its journey. Suvetha et al. (2022) introduced image processing techniques to segregate medical waste. This advanced technology employs imaging to identify the type of waste and categorise it accordingly. It serves as an excellent method to prevent violations in waste segregation. For managing radioactive waste and assessing radioisotope activity, Park et al. (2022) suggested deploying an IoT device on the radioactive container to collect humidity and temperature data. This device gathers real-time information on radioisotope activity and communicates with monitors using digital twin technology.

This paper explores how the Internet of Things (IoT) might be used to efficiently collect and break down biomedical waste. Sensors and other Internet Things devices help hospitals monitor waste cans and the waste they contain in real time (Zhao & Niu 2022). This data can streamline waste collection and disposal procedures, reducing landfill overflow (Karnavel et al. 2023). The decomposition of biological waste can also be monitored with IoT-based technologies (Bamakan et al. 2022). Proper waste disposal and reduced environmental pollution are positive outcomes of studying these data. Biomedical solid waste management solutions built on the Internet of Things can potentially reduce a hospital’s environmental impact (Thaseen Ikram et al. 2023; Wawale et al. 2022). Reducing hospital waste and ensuring that it is disposed of in a safe and ecologically acceptable manner can both be accomplished through the use of IoT to collect, track, and decompose waste in hospitals (Saha & Chaki 2023).

## Methods

The IoT architecture employed for waste bin monitoring is designed to utilise sensor nodes to gather data from waste bins. This innovative system involves the integration of sensors into strategically positioned dustbins in various hospital departments and wards. The data collected from these sensors and actuators is swiftly transferred to the cloud, processed, and analysed in a comprehensive database.

Upon reaching maximum capacity, the smart bins send notifications to the central waste management cloud, triggering an alert to waste collectors for proper collection, segregation, and transportation to a centralised collection facility. These sensor nodes are connected to a wireless Internet system connected to hospital routers that transmit data to a central monitoring station. The ultrasonic sensors incorporated within each bin accurately determine fill levels, ensuring that medical waste can be monitored and disposed of periodically without spillage, thus enhancing the efficiency and safety of the waste management process.

### Study area

**Fig. 1:**
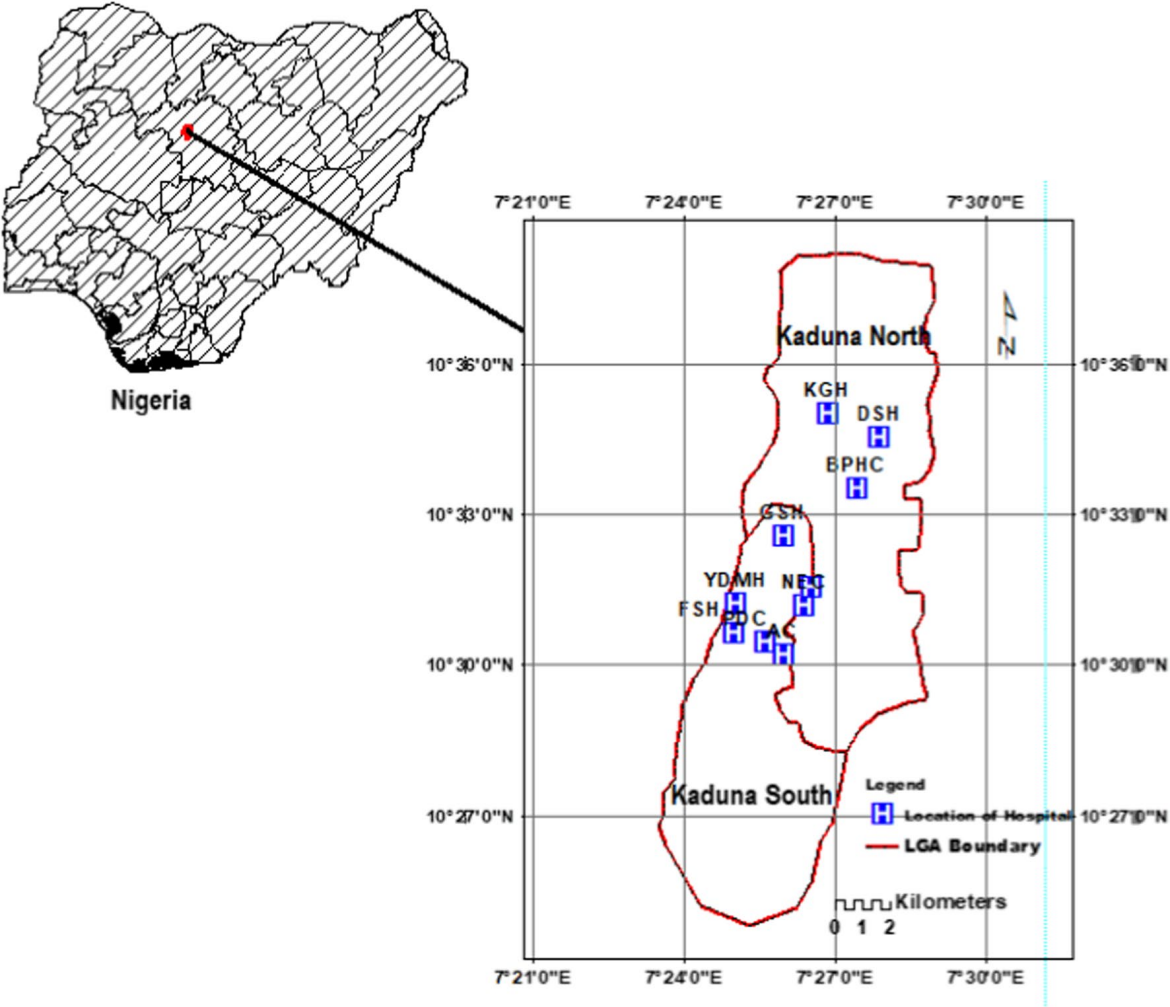
Map showing the location of selected hospitals

The data collection period was held in December 2022 in some selected hospitals in Kaduna metropolis, a northwestern state of Nigeria. A cross-sectional study assessed the biomedical waste generation rate and its management across some selected hospitals in the city. A total of ten (10) hospitals with reliable wireless Internet network availability in the city were selected for the study as shown in Table 1 and Fig. 1.

**Table 1:** The ten selected hospitals and the nature of services provided

Hospital	Abbreviation	Nature of services provided
National Ear Care Centre, Kaduna	NEC	Surgical and special clinical services
Yusuf Dantsoho Memorial Hospital	YDM	General hospital services
Kawo General Hospital	KGH	General hospital services
Giwa Hospital	GH	General hospital services
Alba Clinic & Medical Centre Limited	ACM	General hospital services, excluding paediatric
Fomwan Hospital	FH	Surgical, obstetrics and gynaecology, and special clinical services
Badarawa Primary Health Care Centre	BPH	Medical, obstetrics and gynaecology, and special clinical services
Dialogue Specialist Clinic	DSC	General hospital services, excluding dental services
Primal Diagnostics Centre	PDC	Surgical, medical and special clinical services
Barau Dikko Teaching Hospital	BDT	General hospital services

### Generation of biomedical wastes through the use of IoT technologies

The Internet of Things (IoT) is a network of physical objects embedded with sensors, software, and network connectivity to enable them to collect and exchange data. IoT technologies can improve biomedical waste management in several ways (Qureshi et al. 2023). The following methods were adopted:

- Real-time monitoring of waste: IoT sensors were used to monitor biomedical waste generation, providing real-time information on its status. Ultrasonic sensor–embedded waste bins were strategically placed throughout the healthcare facility. These IoT sensors continuously monitor the fill levels of these bins in real time. To ensure precise and accurate readings, ultrasonic sensors employ the emission of sound waves, which measure the time taken for these waves to rebound and thus determine the fill level. Each waste bin is allotted specific fill-level thresholds, carefully set up by the system. When a bin’s fill level surpasses the established threshold, an event is triggered within the cloud-based system. This information was used to optimise waste collection and disposal and prevent it from being mishandled or disposed of improperly.
- Categorisation of waste: IoT sensors were used to categorise biomedical waste according to its type and hazard level. This was achieved by colour-coding the bins according to each category of waste. Each type of waste is assigned a specific colour code. Green represents organic waste, blue for recyclables, yellow for plastics, and red for hazardous waste. This information was used to ensure that waste is properly disposed of and to prevent the spread of infection.
- Alerts and notifications: IoT sensors were used to send alerts and notifications when biomedical waste reaches a certain threshold. Once the cloud-based system reaches a certain threshold, it generates alerts and notifications. The notifications are received via a specialised monitoring programme for front-end personnel, such as waste collection teams or facility management staff. These alerts include information regarding the location and state of waste bins that need care. Waste collection teams can obtain up-to-date information regarding the condition of waste bins, such as identifying those that are filled and require prompt intervention. The proposed approach optimises waste collection routes by eliminating extra trips and assuring timely waste collection before bin overfilling.
- Data analytics: IoT data were used to analyse biomedical waste generation and disposal patterns. The data obtained from these sensors is wirelessly transferred to a central data hub or gateway within the healthcare facility. This hub is an intermediary connecting the sensors to the cloud-based monitoring system. Subsequently, the data is securely sent to a cloud-based storage and processing platform. Within cloud computing, data is securely stored within a database and subsequently subjected to real-time processing. The processing procedure encompasses the analysis of data, which entails the computation of fill rates, the identification of patterns, and the classification of waste types based on past data. Over a period, the system accumulates a substantial amount of data about waste generation patterns. This data can be analysed to identify trends and optimise waste management processes. This information was used to identify areas where improvements can be made and to develop more efficient waste management strategies.

### System architecture

**Fig. 2:**
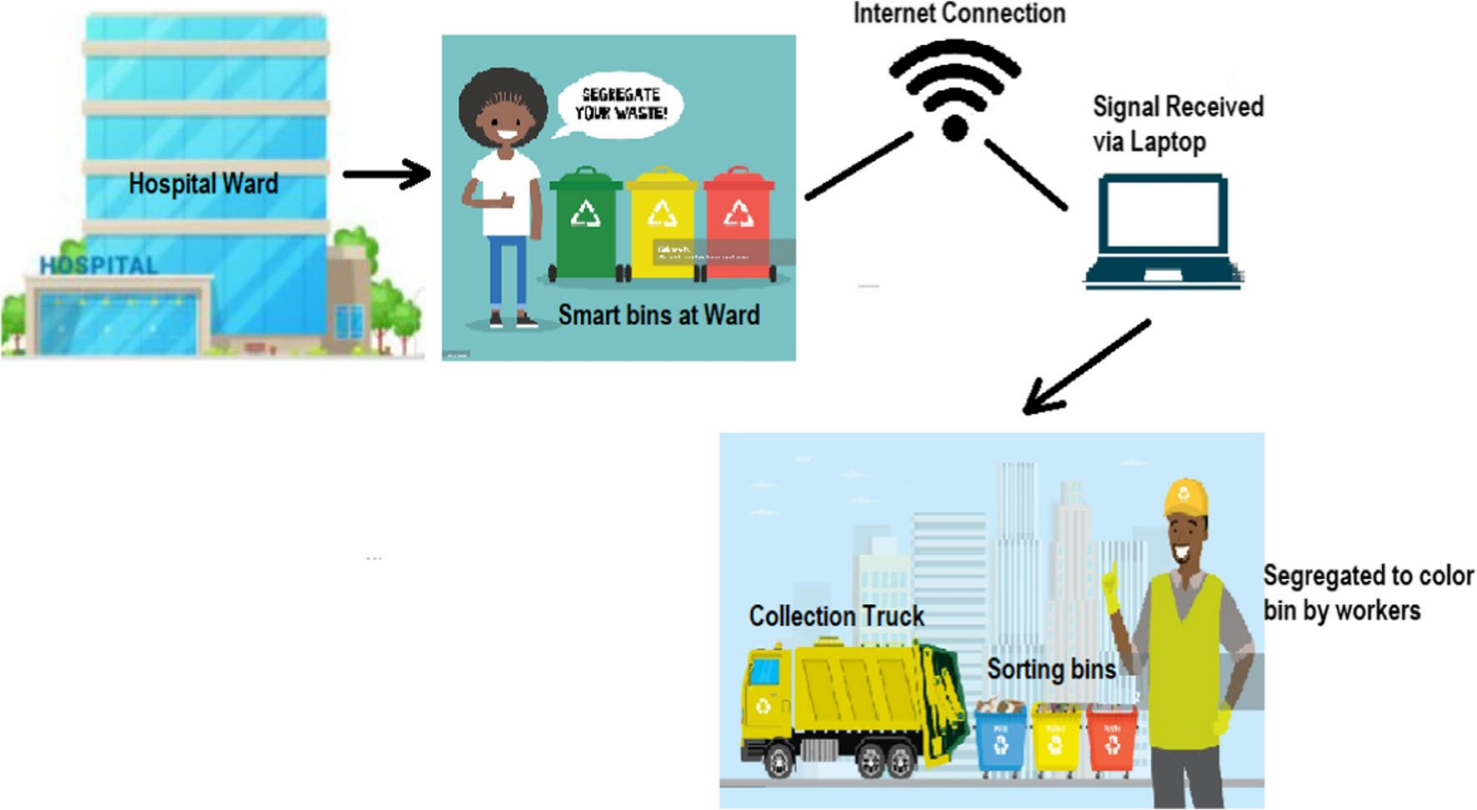
Proposed system architecture

Smart bin technology is a sensor-based system which transmits signals from the point of collection, segregation, transportation, and final disposal. The system architecture is adopted from Vishnu et al. (2021), as described in Fig. 2. The IoT architecture for waste bin monitoring uses end sensor nodes to collect data from waste bins in different wards and departments of the respective hospitals. Bins were all connected to a wireless Internet connection system, which connected to hospital routers and transmitted data to the central monitoring station. Ultrasonic sensors were used in each bin to determine the level of bins when filled. With the help of this system, medical waste can be monitored and periodically disposed of without spillage.

### Smart bin waste management system

This section describes a smart waste management system that uses the Internet of Things (IoT) to collect and sort waste. As Qureshi et al. (2023) described, IoT was utilised for real-time biomedical waste monitoring and collection. Biomedical wastes were collected by state-positioning dustbins equipped with sensors in various hospital departments and wards. The data collected from sensors and actuators were stored in the cloud, processed, and analysed on a database. When the smart bin is full, a notification is sent to the central waste management cloud, which sends an alert to waste collectors for onward collection segregation and transportation of waste to a centralised waste collection facility.

**Fig. 3:**
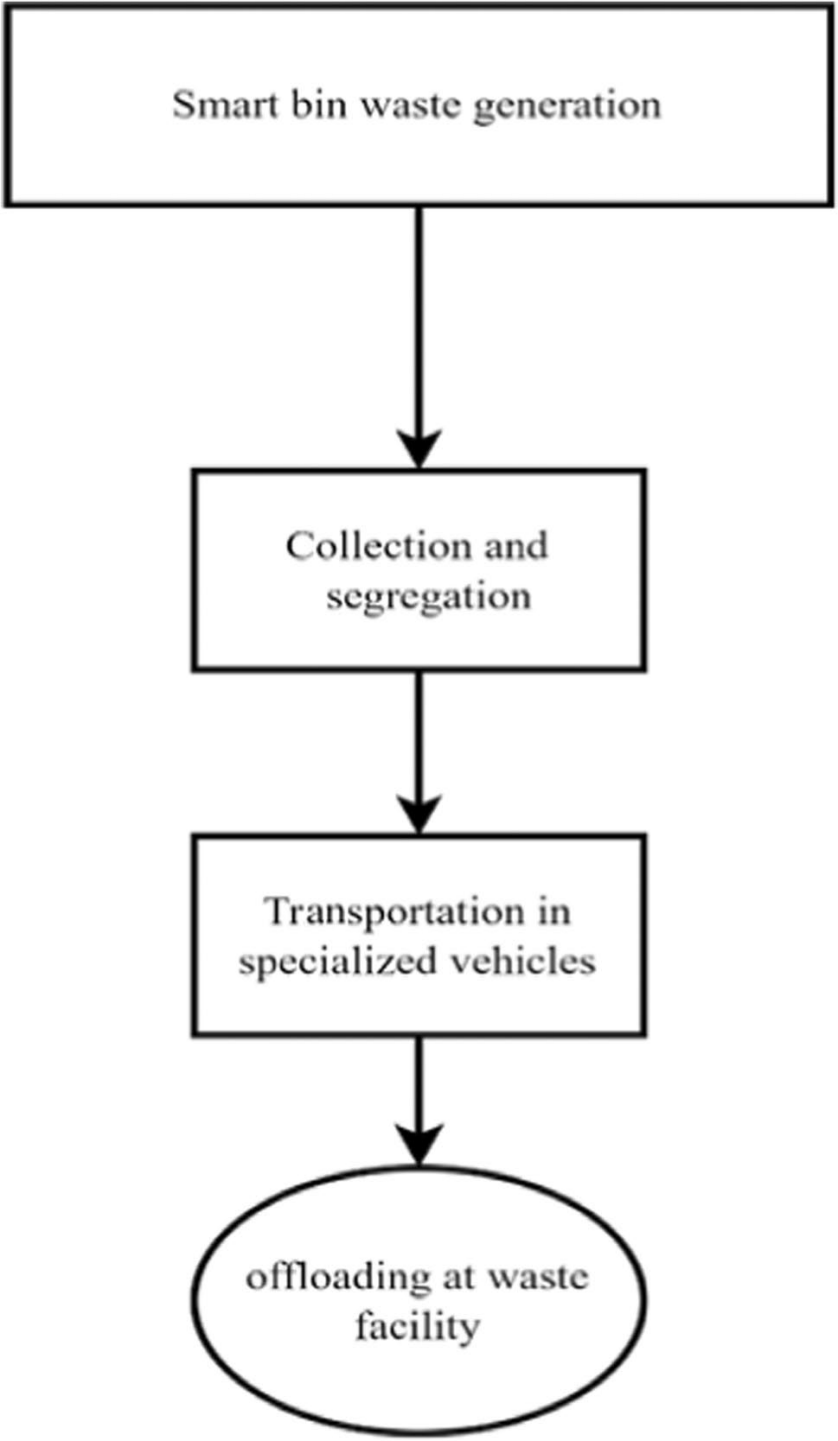
Description of the flow of waste generated

The biomedical waste management process starts with generating waste at the ward/departmental level, basically all waste products of hospital services peculiar to the respective ward. Starting from the source of waste generation, the waste is collected in the smart bin, which sends a notification when full as shown in Fig. 3. A trained waste collector then collects the waste, weighs it, manually segregates and disposes of it into a larger coloured bin in the waste truck/cart according to the different categories of waste, and then transfers it to a waste disposal facility where daily waste generation is assessed.

Key features of the system architecture are as follows:

- Efficient waste sorting: The primary aim of this proposal is to introduce a waste sorting system that is streamlined. Using colour-coding simplifies the waste disposal process for users, making it easier to dispose of waste correctly while maintaining an artistic outlook.
- Smart sensor technology: The proposal integrates cutting-edge sensor technology within the bins, facilitating automated garbage sorting. The proposed sensors can discern the type of discarded trash, diminishing users’ need to manually select the appropriate bin and promoting the maintenance of a sanitary atmosphere.
- Real-time feedback: The idea incorporates a real-time feedback mechanism, such as LED indicators or audible alerts, to promptly notify users of the accuracy of their disposal actions. Prompt feedback serves as an educational tool for users, fostering and reinforcing appropriate waste categorisation practices and promoting a more pristine environment.
- Data collection and analysis: Intelligent waste receptacles can gather data about the various types and volumes of waste. Subsequently, the collected data is transmitted to a centralised system or cloud-based database for analysis. By enabling informed decision-making processes, such as optimising waste collection routes and monitoring recycling rates, this technology contributes to establishing a sustainable environment.
- Customizability: The customisation of the colour-coding system allows for its alignment with local trash categorisation rules and regulations. This ensures that the proposal can be implemented in various regions with different waste management requirements while maintaining an artistic outlook.
- Mobile app integration: The proposal includes a mobile app that complements the smart bins. Front-end workers can use the app to access information on waste disposal and keep track of their progress in keeping the environment clean.
- Remote monitoring and management: Waste management authorities can remotely monitor the fill levels of the smart bins, optimising collection routes, reducing operational costs, and minimising environmental impact. This helps to maintain a sustainable environment.
- Scalability: The system is designed to be scalable, allowing for the gradual implementation of smart bins in various locations within a city or municipality while maintaining an artistic outlook.

## Classifications of biomedical waste

### Data analysis

#### Principal component analysis

This study used 10 sources of hospital solid waste (HSW) to investigate the solid waste generated in the 10 hospitals in the northern part of Nigeria. These sources play a major role in determining the amount of solid waste generated in the hospital (Ishaq et al. 2022). The relationship between each source of solid waste generated in the hospitals is evaluated using the PCA statistical tool. This method involved correlating the existing variables by partitioning/grouping the ones with direct or auto-correlated correlations to identify correlations within the grouped variables (Rodionova et al. 2021). The analysis results determine which variables (type of biomedical waste) are influenced by each other and which can be combined as a single variable. This is different from the known regression modelling, as it does not produce a mathematical relationship between the variables that can be used to produce any of the parameters (Jolliffe 2022). One area of PCA, one of the setbacks of the regression model, does not group the auto-correlated variables; they tend to produce similar results. The regression process, on the other hand, fails to variables that are auto-correlated. An SPSS Software (Version 22.0. Armonk, NY: IBM Corp) was adopted for PCA analysis. In the first step, the data were standardised (*Z*-score method), and then, Kaiser–Meyer–Olkin (KMO) and Bartlett were used to test if factor analysis was applicable (Bucci et al. 2018). This evaluates if the value is within the conventional standard of greater than or equal to 0.5. Table 2 shows the result of the KMO and Bartlett analysis, and it shows that PCA is suitable for the data and was adopted here. A scree plot that shows the main variables was presented, while major components are the principal components with an Eigen value higher than one (Vidal et al. 2016). Subsequently, the key principal component within the variables and the correlations among them and their components were further investigated, using the component plot in the rotated space of the variables. Finally, the regression analysis of the influencing component variable is done to determine the significance of each hospital in generating the study area.

**Table 2:** KMO and Bartlett’s test result

Kaiser–Meyer–Olkin measure of sampling adequacy		.667
Bartlett’s test of sphericity	Approx. Chi-square	105.375
	Df	28
	Sig	.000

## Results and discussions

### Smart bin responses

Table 3 provides valuable insights into the waste management efforts of several hospitals based on the number of smart bins provided, the total number of times the smart bins were filled up (which generated signals), the average number of signals received per smart bin, and the average waste collector’s response time in minutes. The hospitals vary in terms of the services they provide and the number of wards/departments, which determines the number of smart bins in the hospital. Upon analysing the data, it can be seen that the hospitals “BDT”, GH, and YDM have the highest number of smart bins provided (7), evidently because they are general hospital service providers and have 7 distinct wards for each. Hospital NEC has only two wards and has just 2 smart bins at each ward. Hospital BDT received the highest number of filled-up signals (36) from the 7 smart bins provided, which means that they have a higher demand for waste collection services. Despite this, their waste collection response time is impressive, averaging 16 min, which suggests a well-organised waste collection system.

**Table 3:** Responses received from smart bins

Hospital	Location of smart bins	No. of smart bins provided	Total no. of times filled-up signals were received	Average no. of signals received per smart bin	Average waste collector’s response time (min)
NEC	Surgical ward and eye clinic ward	2	11	6	5
YDM	Surgical ward, medical ward, paediatrics ward, obstetrics and gynaecology ward, special clinical services ward, dental ward, delivery ward	7	22	3	14
KGH	Surgical ward, medical ward, paediatrics ward, obstetrics and gynaecology ward, special clinical services ward, delivery ward	6	24	4	11
GH	Surgical ward, medical ward, paediatrics ward, obstetrics and gynaecology ward, special clinical services ward, dental ward, delivery ward	7	21	3	8
ACM	Surgical ward, medical ward, obstetrics and gynaecology ward, special clinical services ward, dental ward, delivery ward	6	23	4	12
FH	Surgical ward, obstetrics & gynaecology ward, special clinical services ward, delivery ward	4	21	5	9
BPH	Medical ward, obstetrics & gynaecology ward, special clinical services ward	2	11	6	7
DSC	Surgical ward, medical ward, paediatrics ward, obstetrics and gynaecology ward, special clinical services ward, delivery ward	6	21	4	8
PDC	Surgical ward, medical ward, special clinical services ward	3	12	4	3
BDT	Surgical ward, medical ward, paediatrics ward, obstetrics and gynaecology ward, special clinical services ward, dental ward, delivery ward	7	36	5	16
		50	176		

On the other hand, the hospital “PDC” provided only 3 smart bins, but interestingly, it received a considerable number of signals (12 times), suggesting that the waste bins in this hospital tend to fill up quickly. This can be attributed to the nature of the services provided in the hospital, such as surgical services, which agrees with Ogbonna (2013), who showed that surgical wards tend to produce significant biomedical waste. Although they have a high average of 4 signals per smart bin, the waste collector’s response time is relatively low at 3 min, indicating a prompt waste disposal process. This may be due to the size of the hospital being the smallest out of the ten selected hospitals, so workers would take a shorter period to get to the location of the bins.

### Rate of waste generation

Data on different hospitals’ waste management practices are included in Table 4. The average number of beds, average daily waste generation, average daily waste per bed, average daily biomedical waste generation, average daily biomedical waste generation per bed, and average daily percentage of biomedical waste are all presented for all ten hospitals.

**Table 4:** Rate of hospital waste generation

Hospitals	Average no. of beds	Average weight of waste (kg/day)	Average weight of waste/bed (kg/bed/day)	Average weight of biomedical waste (kg/day)	Average weight of biomedical waste/bed (kg/day)	Percentage of biomedical waste (%)
NEC	50	94.00	1.64	37.20	0.74	45.49
YDM	150	222.00	1.29	100.44	0.67	52.00
KGH	100	228.00	1.98	100.82	1.01	50.83
GH	75	156.00	1.81	70.34	0.94	51.83
ACM	65	161.20	2.16	73.59	1.13	52.47
FH	55	64.90	1.03	24.77	0.45	43.86
BPH	25	34.50	1.20	13.09	0.52	43.62
DSC	50	64.00	1.11	28.04	0.56	50.36
PDC	100	158.00	1.37	63.09	0.63	45.89
BDT	240	432.00	1.57	180.03	0.75	47.90
Average		161.46	1.52	69.14	0.74	48.43
SD		115.86	0.38	49.62	0.22	3.49

The average number of beds in Table 4 clearly shows how differently sized the hospitals are. For instance, BDT has 240 beds, making it the hospital with the most beds, whereas BPH has 25 beds, making it the hospital with the fewest beds. The difference in bed counts directly affects how much waste is produced. Hospitals like KGH and YDM, which have 228 beds and 222 beds, respectively, tend to produce a higher average weight of waste each day than hospitals with fewer beds. The average weight of waste per day ranges from 34.50 to 432.00 kg, with an overall average of 161.46 kg. Waste per bed each day ranges in weight from 1.03 to 2.16 kg, with an average of 1.52 kg. This result agrees with Gupta and Boojh (2006) and Nwakuwuo et al. (2014), who found that the average daily generation of hospital waste in Owerri, Nigeria, was 1.3 kg per bed, with a range of 1.1 to 2.5 kg per bed. The study by Wahab and Adesanya (2011) found that public hospitals in Ibadan generated between 0.37 and 1.25 kg of waste per patient per day, while private hospitals generated between 0.12 and 0.28 kg per patient per day. In another study (Ogbonna 2013), the average amount of medical waste generated per day in the three categories of hospitals (large, medium, and small) was 17.66 kg, 7.89 kg, and 2.36 kg, respectively.

The proportion of biomedical waste is another crucial factor to consider. Due to the possible risks, managing biomedical waste presents special issues. According to the table, most hospitals have a biomedical waste proportion that ranges from 43.62 to 52.47%. This result shows that the composition of hospital waste in Nigeria varies depending on the type of hospital and is in agreement with Abdullahi et al. (2015).

**Fig. 4:**
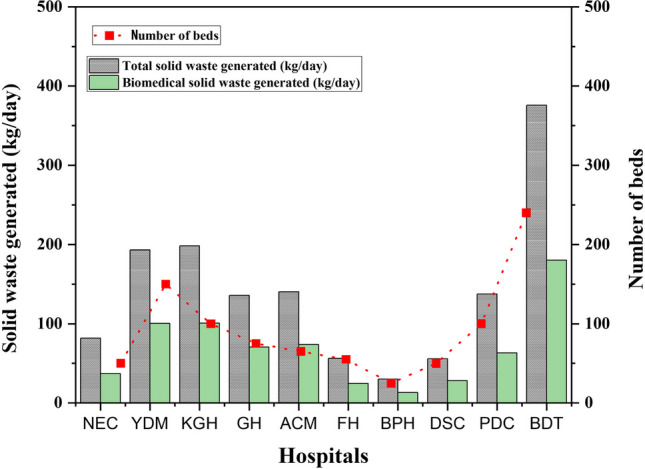
Chart displaying the overall generation of solid waste and biomedical waste

With daily weights ranging from 13.09 to 180.03 kg, the formation of biomedical waste weighs an average of 69.14 kg daily (Fig. 4). Similarly, the weight of biomedical waste per bed per day ranges from 0.45 to 1.13 kg, with an average of 0.74 kg. Biomedical waste is present in hospitals in varying amounts, ranging from 43.62 to 52.47% and an average of 48.43%. The figures in the current study are close to that of Shammi et al. (2021), who found the range of biomedical waste as 35–39% but falls within the range obtained by Aske (2013), which is between 40 to 60% of total hospital waste. The table summarises how the mentioned hospitals differ regarding waste creation and management techniques, greatly influenced by the services provided.

### Distribution of biomedical waste type

Table 4 provides data on various waste types generated by different hospitals, including sharps, chemicals, radioactive materials, pathological waste, infectious waste, pharmaceuticals and genotoxic waste, highly infectious waste, and non-hazardous waste. The values are presented in kilograms per day (kgd^−1^) as percentages of the total waste generated.

Table 5 shows how the quantities and percentages of waste generation types vary across different hospitals. For instance, the highest amount of sharps waste is generated by BDT Hospital, with a value of 10.98 kgd^−1^, while the lowest is from FH Hospital, with 1.21 kgd^−1^; this can be attributed to the difference in sizes of the hospitals in terms of number of beds and also due to the nature of services they provide. General services hospitals such as BDT, YDM, and KGH generally produce more biomedical waste because they are consequently larger, i.e. in terms of the number of beds, and also perform all hospital services. Regarding chemical waste, BDT Hospital again generates the highest amount with 21.06 kgd^−1^, while NEC Hospital generates the least with 3.72 kgd^−1^. YDM and KGH also generate notable quantities of radioactive waste (0.60 kgd^−1^ and 0.50 kgd^−1^, respectively), pharmaceuticals, and genotoxic waste (16.19 kgd^−1^). Because of the significant amounts of hazardous waste that these hospitals produce, these findings imply that they could need specialised waste management and disposal techniques. When considering the overall mean values, we find that the average quantities of each waste type across all hospitals are as follows: sharps (3.02 kgd^−1^), chemicals (7.52 kgd^−1^), radioactive materials (0.37 kgd^−1^), pathological waste (10.72 kgd^−1^), infectious waste (0.44 kgd^−1^), pharmaceuticals and genotoxic waste (10.43 kgd^−1^), highly infectious waste (14.90 kgd^−1^), and non-hazardous waste (18.55 kgd^−1^). Sharps waste appears to have rather stable percentages among hospitals, ranging from 4.00 to 6.10% for specific waste kinds. In contrast, there are a lot of variances among other waste categories. For instance, the range for highly infectious waste is 9.50–27.00%, whereas the range for radioactive waste is 1.51–21.06%. In comparison, the study by Nwakuwuo et al. (2014) found that the majority of hospital waste (72.6%) was classified as non-infectious, followed by infectious waste (22.3%) and hazardous waste (5.1%). Datta et al. (2018) also showed that the most common types of non-infectious waste were food waste (34.2%), paper waste (23.9%), and plastic waste (15.1%), while the most common types of infectious waste were sharps (54.4%), laboratory waste (23.3%), and soiled dressings (17.9%). Hospital size, specialisation, and waste management procedures are a few variables that may impact these variations. This is confirmed by the study by Wahab and Adesanya (2011), who found that the most common type of waste generated in hospitals was infectious waste—accounting for 32.43% of waste in public hospitals and 38.89% in private hospitals. Another study (Ogbonna 2013) found that the percentage of hazardous waste generated was 41% in large hospitals, 35% in medium hospitals, and 18% in small hospitals, which agrees with the findings from this study.

**Table 5:** Distribution of biomedical waste by type

Hospitals	Sharps (kgd^−1^)	Percent	Chemicals (kgd^−1^)	Percent	Radioactive (kgd^−1^)	Percent	Pathological (kgd^−1^)	Percent	Infectious (kgd^−1^)	Percent	Pharmaceuticals & genotoxic (kgd^−1^)	Percent	Highly infectious (kgd^−1^)	Percent	Non-Hazardous waste (kgd^−1^)	Percent
NEC	1.49	4.00	3.72	10.00	0.15	0.40	5.77	15.50	6.21	16.70	10.60	28.50	2.42	6.50	6.85	18.40
YDM	4.52	4.50	12.05	12.00	0.60	0.60	16.07	16.00	27.12	27.00	12.55	12.50	17.58	17.50	9.94	9.90
KGH	3.43	3.40	9.07	9.00	0.50	0.50	12.60	12.50	12.40	12.30	15.63	15.50	10.59	10.50	36.60	36.30
GH	2.74	3.90	7.74	11.00	0.07	0.10	9.50	13.50	15.48	22.00	12.31	17.50	9.50	13.50	13.01	18.50
ACM	1.77	2.40	9.57	13.00	0.59	0.80	10.67	14.50	16.19	22.00	12.14	16.50	12.14	16.50	10.52	14.30
FH	1.21	4.90	1.98	8.00	0.02	0.09	2.48	10.00	6.69	27.00	3.72	15.00	3.59	14.50	5.08	20.51
BPH	0.62	4.70	1.51	11.50	0.01	0.10	2.16	16.50	2.84	21.70	1.96	15.00	1.96	15.00	2.03	15.50
DSC	0.95	3.40	3.79	13.50	0.22	0.80	4.91	17.50	5.80	20.70	3.51	12.50	3.22	11.50	5.64	20.10
PDC	2.52	4.00	4.73	7.50	0.13	0.20	10.41	16.50	13.88	22.00	6.62	10.50	11.04	17.50	13.75	21.80
BDT	10.98	6.10	21.06	11.70	1.44	0.80	29.70	16.50	44.47	24.70	27.90	15.50	26.10	14.50	18.36	10.20
Mean	3.02	4.13	7.52	10.72	0.37	0.44	10.43	14.90	15.11	21.61	10.70	15.90	9.81	13.75	12.18	18.55
SD	3.04	1.00	5.90	2.04	0.44	0.30	8.12	2.31	12.49	4.47	7.61	4.90	7.69	3.44	9.83	7.48

### Waste distribution by ward/department

The distribution of various ward departments across various hospitals is shown in Table 6. The hospitals NEC (21.08%) and YDM (21.53%) contribute the greatest percentages of biomedical waste output in the surgical ward department, reflecting a major emphasis on surgical treatments in these two hospitals. Hospitals KGH (20.35%) and GH (14.45%) are also prominent in this division. While FH (5.05%) hospitals are barely represented, indicating the hospital’s low emphasis on surgical treatment. Overall, surgical services appear well established in all the hospitals studied, with NEC and YDM hospitals dominating this field. A study by Suvenitha and Shalini (2019) agrees with the findings from this study, where it was found that the surgical and gynaecology ward produces the most waste. NEC (56.65%) stands out as having the greatest percentage in the medical ward category, demonstrating a strong focus on medical care. The proportions of YDM (21.43%) and KGH (20.18%) hospitals in this department are especially noteworthy. Hospitals at GH (20.54%) and ACM (20.37%) make comparable contributions. Hospitals at DSC (20.13%) and BDT (20.31%) display a fairly balanced emphasis on surgical and medical wards. Hospitals like FH (20.39%), BPH (25.45%), and PDC (20.04%) also contribute a considerable amount of waste to the medical industry. These figures indicate that most hospitals place a great deal of focus on medical services, with NEC as the main supplier. On the other hand, NEC is a facility for an eye clinic and is anticipated to be more focused on providing medical and surgical services.

**Table 6:** Distribution of waste generation by ward/department

Hospitals	Surgical ward department (kgd^−1^)	Percent	Medical ward department (kgd^−1^)	Percent	Paediatrics ward department (kgd^−1^)	Percent	Obstetrics & gynaecology ward (kgd^−1^)	Percent	Special clinical services (kgd^−1^)	Percent	Dental ward (kgd^−1^)	Percent	Delivery ward (kgd^−1^)	Percent
NEC	21.08	56.65	–	–	–	–	–	–	16.13	43.35	–	–	–	–
YDM	21.53	21.43	26.04	25.93	24.15	24.04	5.70	5.68	8.68	8.64	6.13	6.10	8.21	8.17
KGH	20.35	20.18	26.56	26.34	24.62	24.42	5.82	5.77	8.85	8.78		0.00	14.62	14.50
GH	14.45	20.54	18.45	26.22	17.10	24.31	5.04	7.17	5.77	8.21	4.07	5.79	5.46	7.76
ACM	14.99	20.37	19.34	26.28	–	–	7.80	10.59	11.87	16.13	8.37	11.38	11.22	15.25
FH	5.05	20.39	–	–	–	–	4.89	19.73	5.59	22.59	–	–	9.24	37.29
BPH	–	–	3.33	25.45	–	–	1.94	14.81	4.82	36.83	–	–	–	–
DSC	5.64	20.13	11.61	41.39	7.82	27.90	0.59	2.10	0.90	3.20	–	–	1.48	5.28
PDC	12.64	20.04	12.84	20.35	–	–	–	–	22.89	36.28	–	–	–	–
BDT	36.57	20.31	36.51	20.28	41.40	23.00	13.02	7.23	19.82	11.01	13.98	7.77	18.74	10.41

Table 6 demonstrates significant hospital diversity regarding the department’s waste distribution. This variance could be caused by various elements, including the patient group served, the size and type of hospital, and the accessibility of resources. The table shows that the surgical ward generates the most biomedical waste in most hospitals, followed by the medical ward. Also, the obstetrics and gynaecology ward generates the least waste in most hospitals. The percentage of waste generated in the special clinical services department varies widely due to the contrasting differences in the type of clinical services provided.

Table 7 compares similar studies using IoT in waste management from other locations worldwide.

**Table 7:** Comparison of IoT systems in other locations

Location	IoT technology used	Waste generation	Results	References
Probolinggo City	• Sensors • GPS • A mobile app • A web portal	Municipal solid waste (MSW) generation rate was not determined	• Reduced overflowing bins • Improved efficiency of waste collection • Increased citizen participation • Reduced cost of waste management	Suryaman & Soemarno (2022)
India	• Sensor • Patient tracker app • Wireless communication	Biomedical waste generation rate was not determined	• Minimised human interaction • Automatic waste segregation • Monitoring home-isolated patients	Raundale et al. (2018)
India	• End sensor nodes • LoRaWAN networking • WiFi Cloud server • Graphical user interface (GUI)	MSW generation rate was not determined	• Real-time monitoring of trash bins in public and residential areas • The recycling rate in the city increased by 10%	Vishnu et al. (2021)
Ghana	• Ultrasonic sensors • Servo motor for automatic lid operation • GPS module to determine the bin's location • Remote monitoring GUI	BMW generation rate was not determined	The system successfully detects waste and provides real-time waste level information, which can help prevent medical waste overflow and limit human contact with waste	Annan et al. (2022)
India	• The system uses a Raspberry Pi 4 microcomputer and a camera to capture images of biomedical waste • The images are processed using a machine learning algorithm that was trained on the dataset of 2000 images • The algorithm classifies each image into one of four categories of biomedical waste	• BMW accounts for around 20% of the total waste produced • The system was able to achieve an accuracy of 95% in classifying biomedical waste	The system can effectively segregate hazardous biomedical waste	Suvetha et al. (2022)
India	• A network of sensors • This data is then transmitted to a central server • Machine learning algorithms to predict the amount of garbage that will be generated in the future • The mobile app allows hospital staff to view the data in real time	• The IoT-based system was able to accurately predict the amount of garbage generated in the hospital with an accuracy of 95% • The system helped to reduce the cost of waste management by 20%	The study found that the proposed system was able to accurately predict the amount of garbage generated in the hospital. This information was used by the hospital staff to optimise the collection and disposal of garbage, resulting in a significant reduction in the cost of waste management	Gayathri et al. (2022)
India	• Sensors attached to the waste containers • The sensors collected data on the location, temperature, and humidity of the waste containers • This data was then transmitted to a central server, where it was analysed	The rate of waste generation was not presented in the result	The system was able to track the movement of infectious medical waste in real-time. This information was used to identify potential risks, such as improper disposal of waste containers	Karnavel et al. (2023)

### Statistical results and analysis

The correlation matrix of the PCA between the sorted sources of solid waste is shown in Table 7. It indicates a strong relationship between infective (0.9746), pathology (0.9406), highly infectious (0.9717), and pharmaceutical sources of solid waste, which is explained by the nature and operational procedures of the hospitals (eye centres, teaching, general, and public health centres) during the solid waste collection and sorting. It also indicates the relationship between chemical labs, pathology, and radioactive departments in the hospitals, with both growing during the process, and consequently, their solid waste increases due to the nature of their interrelated activities. There is also a correlation between pharmaceutical solid waste and the highly infectious department (0.8665). The two variables are related to the availability of high-risk patients who need immediate treatment and medication.

In addition to the said relations, other low-significant variables support the clear explanation of the effect of certain variables on the type and sources of solid waste generated in the hospitals. This is supported by the relationship between the sharp and non-hazard sources, common activities/practices in the hospital, and routine processes in all the hospitals. Most hospitals administer drugs and injections more often than radiology and pathological activities, but the waste generated has little impact on the total solid waste in the hospital. This was due to the low number of patients and the citizen’s attitude toward treating minor sicknesses and injuries in private health centres (Louw et al. 2019).

**Fig. 5:**
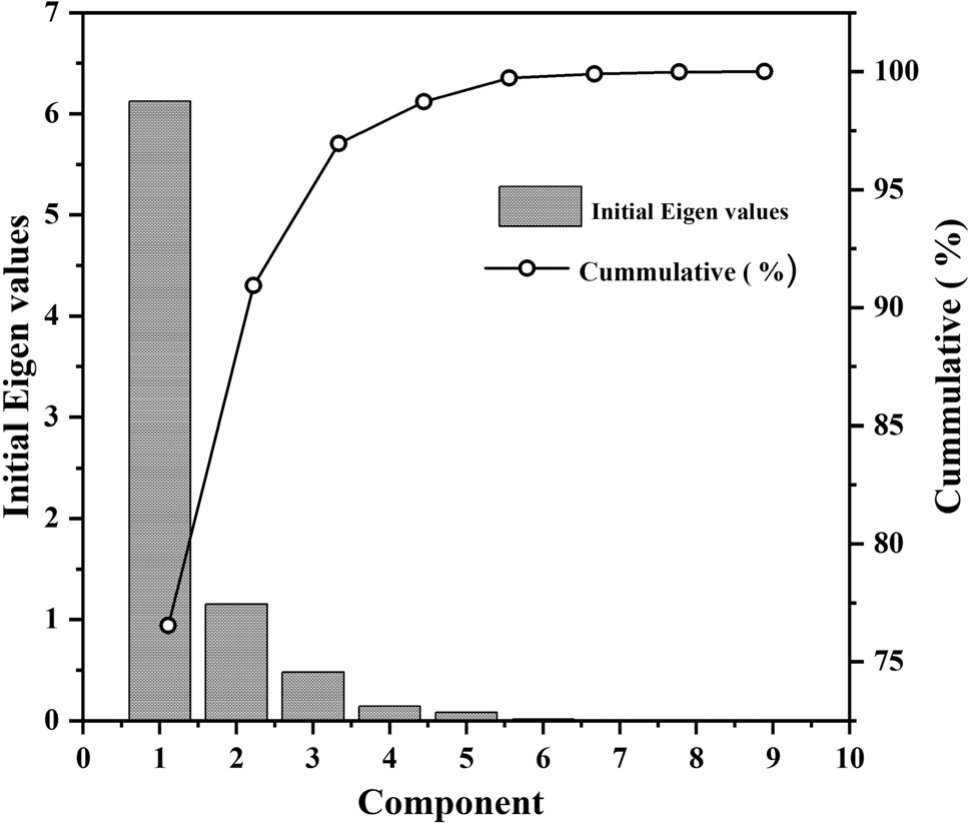
Scree plot of Eigenvalue of PCA

The KMO and Bartlett values show a significant relationship between the identity and correlation matrix. In the analysis, 8 components with their respective eigenvalues are evaluated (Fig. 5 and Table 8). It shows that the values of the eigenvalues summarise the variation of the values accounted for by the component. This is to select the best component that best summarises the overall variation within the whole components in those variables. From Table 9, component one (National Eye Centre (NEC)) will account for much variation of 6.123 of the original measured variables, while component 2 (Yusuf Dantsoho Memorial Hospital (YDM)) accounts for a little variation of 1.152 of the measured variables (see Fig. 1). At the same time, the other component (remaining hospitals) produces trivial value in summarising the variation in the sources of solid waste. It also shows evidence of a two-component solution. Table 7 reflects the impact of the 9 hospitals on the variable measured (sorted solid waste) using the loading criteria of absolute value to attain a measured variable in a given component with much influence.

**Table 8:** Correlation matrix between the sorted solid waste derived from different sources in the hospital

Process	Chemical	Radioactive	Pathology	Infective	Pharmaceutical	Highly_infec	Non_hazard	Sharp
Chemical	1							
Radioactive	0.9559	1						
Pathology	0.9797	0.9406	1					
Infective	0.9684	0.9115	0.9746	1				
Pharmaceutical	0.9446	0.8969	0.9342	0.8821	1			
Highly infectious	0.9615	0.8974	0.9717	0.9794	0.8665	1		
Non-hazard	0.4763	0.4267	0.5008	0.3417	0.5951	0.4505	1	
Sharp	0.3657	0.3733	0.4371	0.5044	0.3421	0.3855	− 0.1101	1

**Table 9:** Categorisation of biomedical waste

Sharps	This waste includes needles, scalpels, lancets, and other sharp objects used in medical procedures. Sharps waste is a biohazard because it can easily puncture the skin and transmit infections (Verma et al. 2008)
Chemicals	This type of waste includes expired or unused drugs, solvents, and other chemicals used in medical settings. Chemical waste can harm human health and the environment, so it is important to dispose of it properly (Jimoh and Abdullahi 2022)
Radioactive	This type of waste includes medical devices contaminated with radioactive materials, such as X-ray machines and radioactive isotopes. Radioactive waste is a serious hazard because it can cause cancer and other health problems (Verma et al. 2008)
Pathological	This type of waste includes human tissues, organs, and body fluids. Pathological waste can be a biohazard because it can contain infectious agents
Infectious	This type of waste includes any waste contaminated with blood or other bodily fluids. Infectious waste can be a biohazard because it can transmit infections such as HIV, hepatitis B, and hepatitis C (Verma et al. 2008)
Pharmaceutical	This type of waste includes expired or unused drugs. Pharmaceutical waste can harm the environment if disposed of improperly (Meleko et al., 2018)
Genotoxic	This type of waste includes chemicals that can damage DNA. Genotoxic waste is a serious hazard because it can cause cancer and other genetic disorders (Verma et al. 2008)
Non-hazardous	This type of waste does not pose any particular hazards. Non-hazardous waste can be disposed of like regular household waste (Verma et al. 2008)

**Fig. 6:**
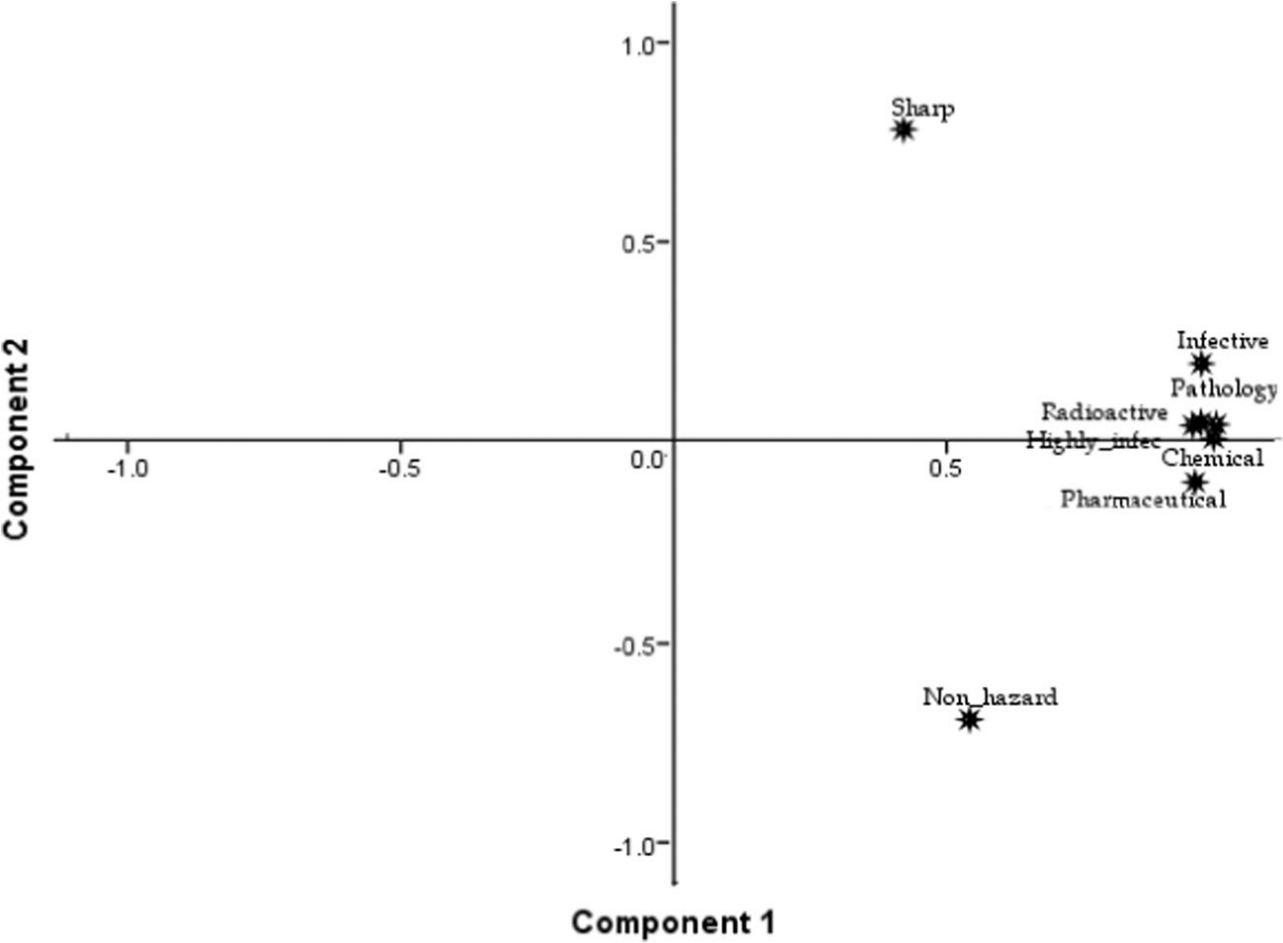
Varimax oblique rotation showing the correlation of the primary components

Figure 6 shows the bi-plot in which the sorted solid waste overlaps the variables. According to PCA, the sorted solid waste has specific factor scores showing the contribution of each sorted waste (measure variable) to each principal component. Thus, sorted solid waste with larger factor scores and subsequently more contribution to the total solid waste generated in the hospital and others had lower eigenvalue and less contribution to it (pathology, chemical, infectious, radioactive, and highly infectious). Equally, some sources had larger factor scores and a higher contribution to component 1 (non-hazardous and sharps), while others (pathology, chemical, infectious, radioactive, and highly infectious) had lower scores and a lower contribution to component 2.

The results obtained in the statistical analyses permit the conclusion of the solid waste management at the hospitals to focus more on six variables sufficient for efficient sorting and management of solid waste in health centres. It anchors more on its merit and demerit of the variables in each one of the six shown in Fig. 6 as follows: (a) Chemical, radiology, pathology, pharmaceuticals, infectious, and highly infectious require highly technical solid waste sorting and disposal training, and therefore, waste generated in those units are usually disposed of in special disposal sites. Burning or industrial recycling is simple and does not require thorough training and skilled personnel; an operator with minimum training can carry it out.

## Conclusion

The research shows that smart bins and other Internet of Things (IoT) technologies have improved biomedical waste (BMW) management, which is a step in the right direction toward solving environmental problems. Ten hospitals with a wireless network and well-placed smart bins provided data showing a 3- to 16-min reaction time between the filled-up alarm and waste pickup. Between 0.45 and 1.13 kg of biomedical waste is generated per hospital bed daily, making up around 48.43% of the total solid waste produced by hospitals. Furthermore, the types of medical services each hospital offers affect the waste distribution by ward. The study shows that infectious and highly infectious wastes comprise a significant proportion of hospital waste in Nigeria. These results demonstrate the promise of the Internet of Things–based waste management technologies for enhancing the effectiveness of healthcare institutions’ management of biological and other types of waste.

## Data Availability

The manuscripts’ data is contained in the text.
